# Long-Term Impact of Tonsillectomy on Quality of Life (QoL) in Patients with Palatine Tonsillitis and Palatine Tonsillar Hypertrophy

**DOI:** 10.3390/jcm14186404

**Published:** 2025-09-10

**Authors:** Aleksander Jurkiewicz, Przemysław Bant, Kornel Szczygielski, Michał Kaczmarczyk, Dariusz Jurkiewicz

**Affiliations:** Department of Otolaryngology and Oncological Laryngology with Cranio-Maxillofacial Surgery Unit, Military Institute of Medicine—National Research Institute, 04-141 Warsaw, Poland

**Keywords:** palatine tonsillar hypertrophy (PTH), palatine tonsillitis (PT), tonsillectomy, quality of life (QoL), Glasgow Benefit Inventory (GBI)

## Abstract

Tonsillectomy is one of the most common surgical procedures and, given the limited efficacy of conservative treatment, remains the primary approach for managing palatine tonsillitis (PT) and palatine tonsillar hypertrophy (PTH). Both conditions negatively affect quality of life (QoL), increase healthcare costs, and contribute to work absenteeism. **Objectives:** The primary aim of this study is to assess QoL following tonsillectomy and uvulopalatopharyngoplasty (UPPP) in patients qualified for surgical treatment due to PT and PTH. **Methods:** A prospective cohort study was conducted among 89 adults (85% follow-up) who had undergone tonsillectomy and UPPP. QoL was assessed 6 years post-op using the Glasgow Benefit Inventory (GBI), the Schwentner questionnaire, and VAS. The study group included 89 patients (85% of those who underwent surgery), including 26 women (29%) and 63 men (71%). **Results:** Patients with PT and PTH showed significant QoL improvement after palatine tonsillectomy. The mean GBI score indicated QoL improvement after both tonsillectomy (+25.7) and UPPP (+15.8), with the most pronounced improvements in physical and general health. Overall, GBI scores were higher in PT compared to PTH (26.6 compared to 15.5), mainly due to better results in the physical health domain (55.9 compared to 12.9). **Conclusions:** Palatine tonsillectomy has a positive impact on QoL in both PT and PTH patients, with higher questionnaire scores observed in the PT group. Tonsillectomy contributed more significantly to the improvement in QoL than UPPP, as measured by the GBI, the Schwentner questionnaire, and the Visual Analogue Scale (VAS). Furthermore, our study demonstrated that, when evaluating and qualifying patients for tonsillectomy, a more comprehensive otolaryngological assessment should be conducted, including evaluation of the nasal cavity and nasopharynx, as comorbid conditions in these regions are correlated with postoperative QoL outcomes.

## 1. Introduction

Diseases of the palatine tonsils contribute to a decreased quality of life [1]. Indications for tonsillectomy have been discussed by a wide group of specialists, and the list of indications for this surgery can be categorized based on their impact on airway patency, infections, and quality of life (QoL) [2]. QoL has been recognized as a significant concept and objective in health and medical research and practice [3]. In recent decades, an increasing number of studies have focused on patients’ QoL, which has led to greater utilization of this parameter [4]. Understanding QoL is important for improving symptom relief, care, and patient rehabilitation. Problems revealed by patient self-reported QoL can lead to significant modifications and improvements in treatment and care as well as may also show that certain therapies provide limited benefit. Such information can be conveyed to future patients to help them anticipate and understand the consequences of their disease and its treatment [5]. In our study, we used the Glasgow Benefit Inventory (GBI), additionally supplemented with the questionnaire used in the study by Schwentner et al. [6].

## 2. Materials and Methods

A prospective cohort study was conducted. A total of 105 subjects meeting the inclusion criteria for the study were selected from among patients qualified for tonsillectomy or uvulopalatopharyngoplasty (UPPP) procedures, which were performed in the operating block of the Department of Otolaryngology and Laryngological Oncology, in conjunction with the Clinical Department of Cranio-Maxillofacial Surgery at the Military Institute of Medicine—National Medical Institute in Warsaw. Patients included 72 men (68.6%) and 33 women (31.4%). The mean age was 42 years for men and 35 years for women. Six years after the surgery, we were able to contact 89 patients (85% of those qualified), including 26 women (29%) and 63 men (71%).

Two patient groups—palatine tonsillitis (PT) and palatine tonsillar hypertrophy (PTH)—were identified based on criteria established in our previous study [7]. Patients qualified for tonsillectomy or UPPP were personally examined by one of the authors of this monograph, thus eliminating preoperative assessment discrepancies. The mean age at the time of surgery in the PTH group was 45.4 years (range: 21–70 years; median: 43.5), and in the PT group it was 35 years (range: 21–70 years; median: 36.5). The majority of participants (53%) were young adults (aged ≤44 years), while middle-aged adults (45–64 years) and older adults (≥65 years) accounted for 36% and 11%, respectively. Age classification was based on the United States Census Bureau definitions [8]. Of the 89 patients, 44 (49%) underwent surgery due to PTH, and 45 (51%) due to PT. Tonsillectomy was performed in 47 patients (53%), and UPPP in 42 patients (47%).

We used the Glasgow Benefit Inventory (GBI) questionnaire as a general patient-reported outcome measure [9]. The GBI is intended for use only once after surgical intervention to assess changes related to a specific medical procedure as a measure of QoL changes [10]. Studies have shown that this questionnaire is specific and appears to be the most sensitive to the impact of otolaryngological interventions, including tonsillectomy and UPPP, in terms of QoL [5]. Furthermore, the GBI is the most commonly used questionnaire in otolaryngology to assess changes in quality of life and benefits after palatine tonsil resection [11,12,13].

Our study was supplemented with additional questions used in the work of Schwentner et al. (Appendix A), which further specify the GBI questionnaire regarding postoperative quality of life after tonsillectomy. In order to validate the questionnaire, we conducted a randomized trial including 15 patients to assess the correct understanding of the text in both English and Polish. Analysis of the responses showed consistent comprehension regardless of the language of the questionnaire. To measure the overall well-being of patients related to palatine tonsil disease, we further applied a visual analogue scale (VAS) ranging from 0 to 10.

We acknowledge the potential for recall bias, as participants reported their experiences retrospectively, and patient-reported outcome measures are inherently subjective. We also recognize that, as an observational study, causal inferences are limited, and while relevant covariates were included to reduce confounding, residual confounding cannot be entirely excluded. Regarding selection bias, we were able to contact 85% of all patients who had been enrolled in the study prior to surgery and subsequently underwent the procedure; the remaining participants were unreachable, declined to participate, or returned incomplete questionnaires, and one patient had died (unrelated to the intervention).

## 3. Results

### 3.1. Overall GBI Results

The overall GBI can be divided into three subscales: general health (GBI_gh_), physical health (GBI_ph_), and social support (GBI_ss_). The observed post-surgery improvement in QoL was primarily attributed to the physical health and general health components, while the social support domain showed relatively minor improvement. Total GBI values were significantly higher in the PT (*M* = 26.6, *SD* = 19.2, *N* = 45) group than in the PTH (*M* = 15.5, *SD* = 14.8, *N* = 44) group (two-tailed *t*-test: t(87) = −3.05, *p* < 0.01, Cohen’s d = −0.65, 95% CI [−1.07, −0.22], indicating a medium effect), primarily due to differences in GBI_ph_ values. The mean GBI_ph_ score was significantly higher in the PT group (*M* = 55.9, *SD* = 40.5, *N* = 45) compared to the PTH group (*M* = 12.9, *SD* = 33.5, *N* = 44) (two-tailed *t*-test: t(87) = −5.45, *p* < 0.01, Cohen’s d = −1.16, 95% CI [−1.60, −0.70], indicating a strong effect). GBI values were also significantly higher in patients who underwent tonsillectomy (*M* = 25.7, *SD* = 19.0, *N* = 48) than in those who underwent UPPP (*M* = 15.8, *SD* = 15.1, *N* = 41) (two-tailed *t*-test: t(87) = 2.69, *p* < 0.01, Cohen’s d = 0.57, 95% CI [0.15, 0.99], indicating a medium effect). A summary of the results is presented in Table 1.

The mean overall GBI value showed an improvement in QoL after both tonsillectomy (+25.7) and UPPP (+15.8). A total of 72 patients had a positive GBI score ranging from +2.78 to +77.78. Ten patients scored 0 on the GBI, including one after tonsillectomy and nine after UPPP (one from the PT group and nine from the PTH group). Seven patients, including four after tonsillectomy and three after UPPP (four from the PTH group and three from the PT group), had negative GBI scores, ranging from –2.77 to –8.33.

Statistically significant differences in GBI scores were found based on patients’ subjective responses. Those who reported no improvement or more frequent episodes of pharyngitis and tonsillitis postoperatively had the lowest GBI scores (*M* = −5.6, *SD* = 2.8, *N* = 3) (ANOVA test: F(3,85) = 16, *p* < 0.01). In contrast, patients who reported “no change” (*M* = 10.1, *SD* = 9.7, *N* = 27) or “slight improvement” (*M* = 16.5, *SD* = 14.6, *N* = 15) exhibited moderate GBI values with no significant differences between the two groups. Patients who reported “significant improvement” achieved the highest GBI (*M* = 31.3, *SD* = 17.1, *N* = 44).

Patients with a history of peritonsillar abscess (*M* = 35.3, *SD* = 18.9, *N* = 13) had significantly higher GBI values than those without such a history (*M* = 18.7, *SD* = 16.7, *N* = 76) (two-tailed *t*-test: t(87) = −3.23, *p* < 0.01, Cohen’s d = −0.97, 95% CI [−1.57, −0.36], indicating a strong effect). Patients who smoked and consumed alcohol (*M* = 14.4, *SD* = 15.4, *N* = 20) had significantly lower GBI scores than those who did not (*M* = 23.1, *SD* = 18.3, *N* = 69), based on a *t*-test (two-tailed *t*-test: t(87) = 1.92, *p* < 0.05, Cohen’s d = 0.49, 95% CI [−0.16, 0.99], indicating a medium effect). Significant differences in GBI scores were also found depending on postoperative swallowing outcomes. Patients who reported no change in swallowing had significantly lower GBI values (*M* = 16, *SD* = 14, *N* = 64) than those reporting moderate improvement (*M* = 33, *SD* = 20, *N* = 11) or significant improvement (*M* = 31, *SD* = 18, *N* = 11) (ANOVA test: F(3,85) = 6.1, *p* < 0.01).

### 3.2. Social Support GBI_ss_ Results

GBI_ss_ values related to social support showed minimal improvement in both the PT (*M* = 5.6, *SD* = 17.8, *N* = 45) and PTH (*M* = 3.4, *SD* = 7.7, *N* = 44) groups. Similar results were observed for the tonsillectomy (*M* = 4.2, *SD* = 15.9, *N* = 48) and UPPP (*M* = 4.9, *SD* = 10.7, *N* = 41) groups. The subgroup of patients who reported the greatest benefit in the social support domain were those with a history of peritonsillar abscess (*M* = 14.1, *SD* = 28.7, *N* = 13), who showed significantly higher GBI scores compared to patients without such a history (*M* = 2.9, *SD* = 8.3, *N* = 76) (two-tailed *t*-test: t(87) = −2.84, *p* < 0.01, Cohen’s d = −0.85, 95% CI [−1.45, −0.25], indicating a strong effect). GBI_ss_ values significantly deviated from a normal distribution, as indicated by the Shapiro–Wilk test (W = 0.37, *p* < 0.05). Notably, 84.27% of patients scored exactly 0 on this subscale. Simultaneously, a one-sample *t*-test revealed that the mean value differed significantly from zero (t(88) = 3.1, *p* < 0.05).

### 3.3. General Health GBI_gh_ Results

GBI_gh_ values were significantly higher among female patients (*M* = 27.9, *SD* = 19.3, *N* = 26) compared to male patients (*M* = 19.4, *SD* = 18.6, *N* = 63) (two-tailed *t*-test: t(87) = 1.92, *p* < 0.05, Cohen’s d = 0.49, 95% CI [−0.01, 0.91], indicating a weak effect).

### 3.4. Physical Health GBI_ph_ Results

GBI_ph_ values for women (*M* = 62.8, *SD* = 38.7, *N* = 26) were significantly higher than for men (*M* = 23.0, *SD* = 39.2, *N* = 63) (one-tailed *t*-test: t(87) = 4.37, *p* < 0.01, Cohen’s d = 1.01, 95% CI [0.54, 1.50], indicating a strong effect). There was a negative weak correlation (r(87) = −0.35, *p* < 0.01) between GBI_ph_ and BMI of patients. Patients with a history of peritonsillar abscess (*M* = 76.9, *SD* = 33.7, *N* = 13) had significantly higher GBI_ph_ values than those without such a history (*M* = 27.4, *SD* = 40.2, *N* = 76) (two-tailed *t*-test: t(87) = −4.19, *p* < 0.01, Cohen’s d = −1.26, 95% CI [−1.87, −0.64], indicating a strong effect).

### 3.5. Comparison of the GBI Questionnaire and Its Schwentner-Modified Version

Values of the parameters in the Schwentner-modified GBI questionnaire were evaluated according to the scale of the GBI questionnaire. Significant differences in the distribution of responses within specific patient groups were tested using Fisher’s exact test or the chi-square test (χ^2^), with a significance level set at *p* < 0.05. The results are presented in Table 2, Table 3, Table 4, Table 5, Table 6, Table 7, Table 8 and Table 9.

### 3.6. Gender-Related Comparison of the GBI Questionnaire and Its Schwentner-Modified Version

The conducted study revealed statistically significant differences in responses between women and men, with women reporting greater improvement in various health aspects following surgery compared to men. The evaluation of individual responses from the GBI and Schwentner-modified questionnaires is presented in Table 3. The overall GBI scores, subscale results by gender, and VAS scores are summarized in Table 4.

### 3.7. Comparison of the PT and PTH Groups in the GBI Questionnaire and Its Schwentner-Modified Version

This study revealed statistically significant differences in response distribution between the PT and PTH patient groups, particularly regarding the frequency of infections, pain intensity, number of follow-up visits to the general practitioner, number of postoperative infections, and the amount of medication taken after surgery. The results are presented in Table 5.

### 3.8. Comparison by Type of Surgery (Tonsillectomy vs. UPPP) in the GBI Questionnaire and Its Schwentner-Modified Version

In the conducted study, statistically significant differences (*p* < 0.05) were found in the distribution of responses between patients who underwent tonsillectomy and those who underwent UPPP. These differences concerned the frequency of recurrent tonsillitis, the severity of throat pain, the impact of surgery on overall life, the number of follow-up visits to a general practitioner, the number of infections, and the amount of medication taken postoperatively. Summary results are presented in Table 6.

### 3.9. Anatomy of the Soft Palate in the GBI Questionnaire and Its Schwentner-Modified Version

Patients with a normal soft palate reported significantly better outcomes compared to those with a flaccid soft palate. These included a lower incidence of tonsillitis, reduced throat pain, greater improvement in overall quality of life, fewer visits to general practitioners, fewer postoperative infections, reduced need for medications, and increased social engagement. Detailed results are shown in Table 7.

### 3.10. Age-Related Comparison of the GBI Questionnaire and Its Schwentner-Modified Version

The study revealed statistically significant differences in response patterns depending on patient age. Young adults reported less postoperative throat pain compared with middle-aged and older patients. In contrast, older adults demonstrated increased self-confidence after surgery compared with both middle-aged patients and young adults. Moreover, older patients more frequently reported a greater sense of care from others after the procedure than middle-aged patients and young adults. The aggregated results are presented in Table 8.

### 3.11. BMI in the GBI Questionnaire and Its Schwentner-Modified Version

Patients who reported no reduction in the number of infections or in medication use after surgery had a significantly higher body mass index (BMI) compared to those who indicated a decrease in the frequency of colds and infections as well as reduced medication use postoperatively. Additionally, the mean BMI for men (M = 30.12, SD = 4.94, N = 63) was significantly higher than that of women (M = 24.35, SD = 5.44, N = 26), *p* < 0.05.

### 3.12. Adenoidectomy in the GBI Questionnaire and Its Schwentner-Modified Version

The analysis of response distribution revealed statistically significant differences in QoL improvement between patients who had undergone adenoidectomy and those who had not. Patients with a history of adenoidectomy reported significantly greater improvement in QoL following tonsillectomy/UPPP compared to those without prior adenoidectomy (*p* < 0.05).

### 3.13. Nasal Patency in the GBI Questionnaire and Its Schwentner-Modified Version

The results indicate that patients with impaired nasal patency (partial or complete) experienced greater improvement in quality of life after surgery, while patients with normal nasal patency reported less improvement or no change. Only 57.1% of patients with normal nasal patency reported significant improvement in quality of life postoperatively, whereas 42.9% of this group indicated no change in overall life after the procedure. The results are presented in Table 9.

### 3.14. VAS Values in Relation to the Type of Surgery and Clinical Diagnosis of PT or PTH

The mean VAS score for patients after tonsillectomy was 8.6 (min = 5.0, max = 10.0, SD = 1.6), and after UPPP it was 8.2 (min = 5.0, max = 10.0, SD = 1.8). The mean VAS score for the PT group was 8.6 (min = 5.0, max = 10.0, SD = 1.7), while for the PTH group it was 8.1 (min = 5.0, max = 10.0, SD = 1.7). There was a positive moderate correlation between GBI and VAS (r(87) = 0.54, *p* < 0.01). The results are presented in Table 10.

### 3.15. Additional Author-Designed Questions

Among the surveyed patients, as many as 88.8% would undergo the same surgery again knowing all the benefits and complications. Those who would not choose to have the surgery again showed significantly lower GBI scores, mainly in the general health (GBI_gh_) and physical health (GBI_ph_) subscales. Additionally, 74.2% of patients expressed willingness to undergo the surgery again if it were necessary as part of private treatment. On the other hand, 20% of those who would refuse private treatment cited financial reasons as the main factor influencing their decision.

## 4. Discussion

Based on retrospective studies, it is believed that up to 90% of adult patients with chronic tonsillitis benefit from tonsillectomy [14]. Our study, based on the GBI questionnaire, confirms these findings—84.5% of patients in the chronic tonsillitis group reported fewer infections after surgery, 83.3% experienced a reduction in the number of tonsillitis episodes, 75.3% reported an improved quality of life, 64.5% reduced their medication use, 66.3% had fewer episodes of pharyngitis, and 56.3% of respondents experienced fewer visits to their family doctor after tonsillectomy. Additionally, 76.5% of participants reported feeling better after the surgery.

Bhattacharyya et al. found no correlation between GBI values and the duration of patient follow-up, suggesting that the benefits of tonsillectomy are durable and long-lasting. However, the average follow-up time in their study was only about 3.5 years [10]. Available publications focus on patient assessments from 6 months to 3 years after surgical intervention [10,15,16,17]. In other similar QoL studies, although conducted shortly after surgery, the response rate varied significantly from 26% [16] to 89%, but these studies had relatively small sample sizes (62 patients) and short follow-up periods (only 6 months) [16]. Based on available data, it can be concluded that the improvement in QoL observed one year after surgery is stable and lasting, as confirmed by numerous studies [18,19]. In our case, the survey was conducted as late as 6 years after the procedure, allowing for an evaluation of the long-term treatment effects from the patients’ perspective. Despite the long interval since surgery, we managed to contact 93 out of 105 patients, with 89 participating in the study (85%). The participation rate was significantly higher than in other similar studies [6,11,20]. Currently, there are no clear guidelines on the optimal timing for assessing long-term GBI outcomes after surgical interventions involving the palatine tonsils. However, a six-year period appears to allow for the most accurate assessment of benefits and complications experienced by patients after tonsillectomy and UPPP, while also preventing patients from forgetting the symptoms they suffered before surgery, thus enabling a more precise evaluation of treatment effectiveness [21].

**Figure 1:**
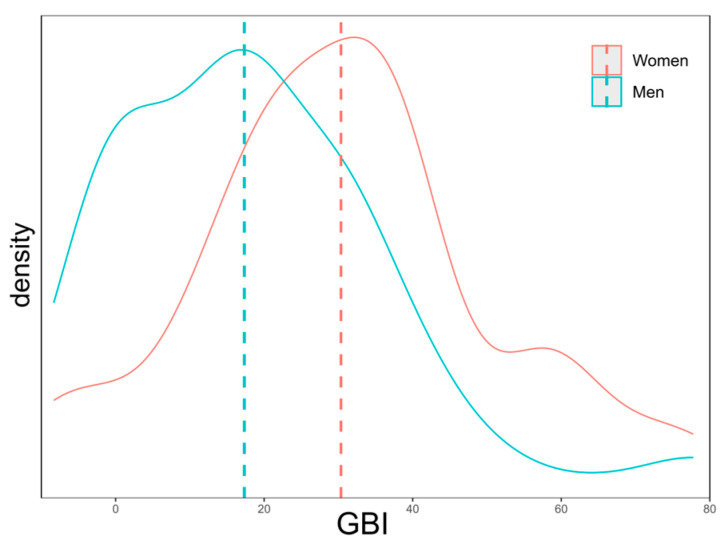
Distribution of responses on the GBI scale by sex.

Compared to other similar studies, where GBI scores for men and women were comparable [6,22], we observed significant discrepancies in the values. Women reported significantly greater improvements in quality of life across many questions in the GBI questionnaire as well as in additional assessments (Figure 1). Men, on the other hand, more frequently than women reported no changes after the surgery.

Gloria Corredor-Rojas and coworkers, studying QoL after rhinologic surgeries, found no correlation between the VAS and GBI scales [23]. In our literature review, we found no studies that utilized the VAS scale and the GBI questionnaire concerning tonsillectomy and UPPP. The high VAS values reported by our patients in both the PT and PTH groups (9.1 and 8.6, respectively) indicate high patient satisfaction with the treatment outcomes and also confirm the long-lasting nature of the improvement in their QoL after the procedure. Furthermore, analysis of the VAS results obtained in our study, compared to the GBI questionnaire results, provides a basis for recommending its use in future research. The VAS scale allows for capturing the overall, subjective patient assessment of the course and effects of the procedure, rather than only selected aspects covered by the GBI questionnaire, which is also used to assess quality of life in patients after other laryngological procedures. Therefore, VAS may serve as a more adequate tool for evaluating overall patient satisfaction and the effectiveness of the intervention from the perspective of the treated individual.

Similar to the studies by Bauman et al. and Richards et al., younger patients (under 30 years of age) showed a statistically significantly greater improvement in quality of life compared to older patients [23,24]. Our results confirm these findings. It has been found that younger adult patients perceive greater benefits from surgical treatment than older patients [23], and this benefit is independent of the surgical technique used [6]. In our study, as many as 70.2% of young adult participants reported a reduction in throat pain after surgery, while in the middle-aged group, this percentage was 53.1%, and only 20% of older patients noticed an improvement in terms of reduced throat pain following the procedure. These results indicate a significant correlation between age and the effectiveness of surgery in alleviating symptoms, suggesting that younger individuals may experience greater relief after the operation. However, older patients demonstrated significantly greater self-confidence after surgery and also reported a notably higher sense of care from others postoperatively. This may result from the greater life experience of this group, more balanced expectations regarding treatment outcomes, as well as more intense social support, which older individuals often receive from their families and loved ones due to their age.

Schwentner et al. reported that improvement in GBI scores among patients after tonsillectomy with coexisting chronic diseases was lower than in patients without chronic diseases [6]. Similarly, in a review, N. Andreou and coworkers concluded that patients undergoing tonsillectomy with chronic conditions will likely derive less benefit than those without chronic diseases [25]. We obtained different results—patients with chronic diseases experienced slightly greater benefits (*M* = 22.28) than patients without chronic diseases (*M* = 19.90). This difference may be due to the fact that our study assessed QoL after both tonsillectomy and UPPP.

So far, we have found no studies addressing the relationship between QoL after tonsillectomy and UPPP and BMI. However, in the analyzed patient group, it was observed that individuals with higher BMI showed slightly less improvement in QoL after the procedure. This difference was particularly noticeable in the GBI questionnaire responses related to the reduction in frequency of colds and infections after surgery, as well as the decreased need for medication. This may be associated with generally lowered immunity and more frequent chronic inflammatory conditions in patients with excessive body weight, which can affect the subjective assessment of the procedure’s effectiveness. Preoperative BMI analysis may represent an important predictive factor for treatment effectiveness, and ignoring the impact of BMI on QoL after tonsillectomy and UPPP should be considered a significant limitation in interpretation. Including BMI in the evaluation of treatment outcomes may allow for a more precise analysis of factors influencing QoL improvement after laryngological procedures and enable better tailoring of postoperative care to individual patient needs.

Similarly, no available studies have addressed the correlation between QoL after tonsillectomy and a prior history of adenoidectomy. Analysis of response distribution in our study revealed that patients with a history of adenoidectomy reported significantly greater improvement in QoL following tonsillectomy and UPPP compared to those without such a history. This may result from previous positive experiences with surgical treatment in the oral and pharyngeal area, as well as greater familiarity with potential postoperative pain and a more informed attitude toward the recovery process. Additionally, the more noticeable improvement in overall breathing comfort after the procedure may play an important role. Furthermore, prior positive surgical experiences may have influenced patients’ subjective expectations and their assessment of the surgery’s effectiveness.

An interesting and original finding in our study, previously omitted in the literature, was the difference in patient response distribution depending on the degree of nasal patency, assessed preoperatively by one of the researchers. In no available studies were patients undergoing tonsillectomy simultaneously subjected to a detailed rhinological evaluation, which makes our observations unique. The results indicate that patients with greater nasal obstruction reported significantly greater improvement in QoL after surgery, as reflected in the GBI extended with the Schwentner questionnaire and the VAS scale, despite the procedure not directly affecting nasal patency. This may be related to a subjective sense of overall improved respiratory function following surgical intervention, as well as a possible synergy between improved pharyngeal patency and perceived breathing comfort. No data regarding the correlation between quality of life after tonsillectomy and the degree of nasal patency were found in the available sources, highlighting the need for further targeted research in this area.

**Figure 2:**
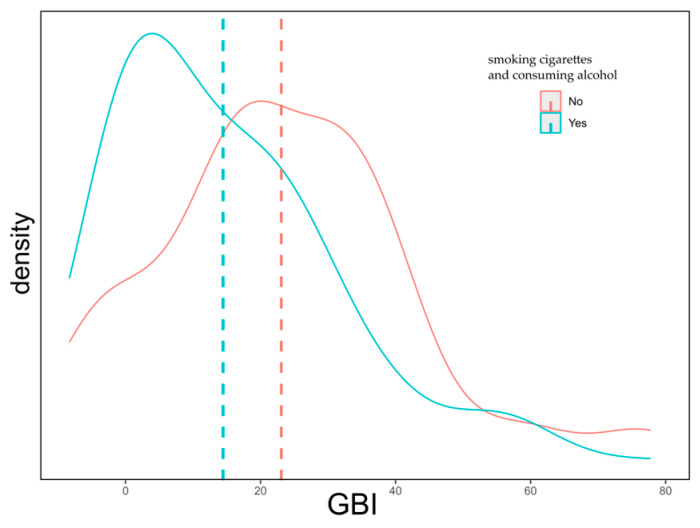
Distribution of responses on the GBI scale according to cigarette smoking and alcohol consumption.

Koskenkorova et al. did not observe significant differences in GBI questionnaire scores between smokers and non-smokers [16]. However, it is not surprising that patients included in our study who smoked cigarettes and consumed alcohol achieved significantly lower scores on the GBI (*M* = 14.44) scale compared to non-smokers and non-drinkers, who scored higher on the GBI (*M* = 23.07) scale (Figure 2). This may be due to a generally lower level of physical and mental health in this group, which most likely negatively affects the subjective assessment of postoperative quality of life and limits the body’s ability to regenerate. Additionally, it should be assumed that individuals who smoke and consume alcohol tend to lead a less hygienic lifestyle and take less care of their health, which may also significantly impact treatment outcomes and the subjective evaluation of its effectiveness.

Bhattacharyya et al., using the GBI scale, also documented an improvement in QoL after tonsillectomy in patients with chronic tonsillitis, along with a reduction in the need for antibiotics, doctor visits, and work absenteeism [20]. Senska et al. reported similar findings regarding improved QoL and decreased demand for healthcare services [26]. Therefore, it is reasonable to expect that tonsillectomy will lead to a significant reduction in healthcare utilization and work-related absences. This improvement in healthcare and economic outcomes following tonsillectomy results in cost savings, as tonsillectomy is considered an “investment” in the anticipated improvement in health status. The cost recovery from tonsillectomy is achieved through savings on medication expenses, doctor visits, and work-related costs [20]. The lower number of infections, sore throat episodes, reduced medication use, and fewer visits to the family doctor observed in our study six years after surgery confirm all of the above findings, taking into account the long time elapsed since the operation.

An issue not previously addressed in the literature, but potentially significant in evaluating the value of surgical interventions, is patients’ willingness to undergo the procedure again. The results of our study indicate that as many as 88.8% of patients, aware of both the benefits and complications, would choose to have the surgery again. This is particularly important considering that this decision takes into account the patient’s prior experience with the procedure, including postoperative pain, hospitalization, preoperative stress, and other inconveniences. Although the GBI questionnaire results did not always unequivocally indicate an improvement in QoL after surgery, the declaration of such a high percentage of patients clearly supporting repeat surgery speaks to the significant, subjectively perceived therapeutic value of the intervention. Such a response may therefore constitute an important complement to quantitative data and highlights the need to include the patient’s perspective in a comprehensive assessment of treatment effectiveness. Elysia Grose and coworkers found that only 21% of patients who had previously undergone tonsillectomy or septoplasty would be willing to pay the anticipated cost of these surgeries if they were not covered by government health insurance [27]. In our study, as many as 74.2% of patients declared that, knowing all the benefits and potential complications associated with tonsillectomy and UPPP, they would decide to undergo these procedures again even if they had to pay privately. This result is entirely justified given the significant improvement in patients’ QoL after the surgeries, as reflected in results from analysis of the GBI, Schwentner-modified GBI, and VAS questionnaires, as well as the clear relief from symptoms experienced before the intervention. This suggests a high perceived value of these procedures from the patients’ perspective.

## 5. Conclusions

In conclusion, we showed that tonsillectomy improves QoL in both groups of patients with palatine tonsillitis (PT) and palatine tonsillar hypertrophy (PTH), with a higher effect observed in the PT group. Analysis of the results from GBI, Schwentner-modified GBI, and VAS questionnaires indicates that tonsillectomy has a greater positive impact on QoL than UPPP. Furthermore, our studies demonstrated that patient evaluation and qualification for tonsillectomy should include a more comprehensive laryngological assessment, such as nasal and nasopharyngeal evaluation, since other conditions in the nasal and nasopharyngeal area correlate with postoperative QoL.

## Figures and Tables

**Table 1:** Mean values and standard deviations (in parentheses) of GBI and its subscale values as well as VAS for all patients, patients classified to the PT or PTH group, and patients qualified for tonsillectomy or UPPP.

Scale	Overall Result	PT	PTH	Tonsillectomy	UPPP
Overall GBI	21.1 (17.9)	26.6 (19.2)	15.5 (14.8)	25.7 (19.0)	15.8 (15.2)
GBI_gh_	21.9 (19.1)	24.5 (20.2)	19.2 (17.7)	24.9 (19.7)	18.4 (17.9)
GBI_ss_	4.5 (13.7)	5.6 (17.8)	3.4 (7.7)	4.2 (15.9)	4.9 (10.7)
GBI_ph_	34.6 (42.9)	55.9 (40.5)	12.9 (33.5)	50.4 (43.6)	16.3 (34.3)
VAS	8.4 (1.7)	8.6 (1.7)	8.1 (1.7)	8.6 (1.6)	8.2 (1.8)

**Table 2:** Patients’ evaluation of surgical treatment outcomes according to selected items from the GBI and Schwentner questionnaires.

Criteria	Percentage of Patients (%)
Improved quality of life	75.3
Better overall well-being	76.5
Fewer health-related inconveniences	48.3
Increased optimism about the future	42.7
Fewer episodes of tonsillitis	66.3
Fewer infections	59.6
Less sore throat pain	58.5

**Table 3:** Evaluation of surgical treatment outcomes by gender based on selected items from the GBI and Schwentner questionnaires.

Criteria	Percentage of Women (%)	Percentage of Men (%)
Fewer primary tonsillitis episodes	96.2	54.0
Fewer infections	92.3	46.1
Less throat pain	80.7	49.2
Improved overall QoL	84.6	71.5
Significant improvement in QoL	65.4	30.2
Moderate improvement in QoL	19.2	41.3
Fewer doctor visits	69.3	22.2
No change in the number of doctor visits	23.1	69.8
Reduction in the number of medications	73.1	31.8
No change in medication use	19.2	63.5
Reduction in swallowing problems	38.5	19.0

**Table 4:** Gender-related mean values and standard deviations (in parentheses) of GBI and its subscale values as well as VAS for all patients, patients classified to the PT or PTH group, and patients qualified for tonsillectomy or UPPP.

Scale	Overall Result		PT		PTH		Tonsillectomy		UPPP	
	Women	Men	Women	Men	Women	Men	Women	Men	Women	Men
Overall GBI	30.3 (19.0)	17.3 (16.2)	30.8 (19.7)	22.9 (18.4)	28.3 (13.9)	13.9 (13.8)	31.4 (18.9)	20.4 (17.9)	22.2 (21.7)	15.3 (14.8)
GBI_gh_	27.9 (19.3)	19.4 (18.6)	28.4 (20.0)	21.2 (20.2)	25.8 (17.8)	18.4 (17.8)	28.8 (19.1)	21.3 (20.0)	20.8 (23.2)	18.2 (17.8)
GBI_ss_	7.7 (21.2)	3.2 (8.9)	7.1 (22.7)	4.2 (12.3)	10.0 (14.9)	2.6 (6.1)	6.5 (21.8)	2.0 (7.3)	16.7 (16.7)	4.0 (9.8)
GBI_ph_	62.8 (38.7)	23.0 (39.2)	64.3 (35.9)	48.6 (43.7)	56.7 (53.5)	7.3 (26.2)	66.7 (35.2)	35.3 (45.7)	33.3 (60.1)	14.9 (32.4)
VAS	9.3 (0.9)	8.0 (1.8)	9.2 (0.9)	8.1 (2.0)	9.6 (0.9)	8.0 (1.7)	9.3 (0.9)	7.9 (1.9)	9.3 (1.2)	8.1 (1.8)

**Table 5:** Evaluation of surgical treatment outcomes by groups of patients with palatine tonsillitis (PT) and palatine tonsillar hypertrophy (PTH) based on selected items from the GBI and Schwentner questionnaires.

Criteria	PT (%)	PTH (%)
Fewer primary tonsillitis episodes	93.3	38.7
No change in the number of tonsillitis episodes	4.40	56.8
Less throat pain	82.2	34
No change in throat pain levels	38.2	61.4
Reduced the number of infections	84.5	34.1
No change in the number of infections	11.1	56.8
Fewer doctor visits	62.8	9.1
No change in the number of doctor visits	31.1	81.8
Reduction in the number of medications	64.5	22.8
No change in medication use	31.1	70.5

**Table 6:** Evaluation of surgical treatment outcomes by type of surgery based on selected items from the GBI and Schwentner questionnaires.

Criteria	Tonsillectomy (%)	UPPP (%)
Fewer primary tonsillitis episodes	83.3	46.3
No change in the number of tonsillitis episodes	18.2	51.2
Less throat pain	75.0	39
Improvement in QoL	85.4	63.4
Significant improvement in QoL	54.2	24.4
Moderate improvement in QoL	31.2	39
No improvement in QoL	14.6	31.7
Fewer visits to the general practitioner	56.3	12.2
Significant reduction in visits	41.7	9.8
Moderate reduction in visits	14.6	2.4
No change in the number of visits	35.4	80.5
Fewer infections after surgery	77.1	39.0
Significant reduction in infections	52.1	19.5
Moderate reduction in infections	25.0	19.5
No change in the number of infections	18.8	51.2
Reduction in the number of medications	58.3	26.8
Significant reduction in medication use	35.4	212.2
Moderate reduction in medication use	22.9	14.6
No change in medication use	35.4	68.3

**Table 7:** Evaluation of surgical treatment outcomes by anatomy of the soft palate based on selected items from the GBI and Schwentner questionnaires.

Criteria	Normal Soft Palate (%)	Flaccid Soft Palate (%)
Fewer primary tonsillitis episodes	81.4	52.2
Less throat pain	72.1	45.7
Improvement in QoL	81.4	69.6
Significant improvement in QoL	55.8	26.1
Moderate improvement in QoL	25.6	43.5
No improvement in QoL	16.3	28.3
Fewer visits to the general practitioner	51.2	21.7
Significant reduction in visits	41.9	13.0
Moderate reduction in visits	9.3	8.7
No change in the number of visits	44.2	67.4
Fewer infections after surgery	73.1	47.8
Significant reduction in infections	53.5	21.7
Moderate reduction in infections	18.6	26.1
No change in the number of infections	25.6	41.3
Reduction in the number of medications	58.1	30.4
Significant reduction in medication use	37.2	13.0

**Table 8:** Evaluation of surgical treatment outcomes by age group based on selected items from the GBI and Schwentner questionnaires.

Criteria	Young Adult (%)	Middle Adult (%)	Older Adult (%)
Less throat pain	70.2	53.1	20.0
Increased self-confidence	14.9	25.0	50.0
Greater sense of care from others	2.1	15.6	20.0

**Table 9:** Evaluation of surgical treatment outcomes by nasal patency based on selected items from the GBI and Schwentner questionnaires.

Criteria	Complete Impaired Nasal Patency (%)	Partial Impaired Nasal Patency (%)	Normal Nasal Patency (%)
Improvement in QoL	81.4	75.0	57.1
Significant improvement in QoL	41.9	31.2	57.1
Moderate improvement in QoL	39.5	43.8	0.0
No improvement in QoL	16.3	21.9	42.9

**Table 10:** VAS values for patients classified to the PT or PTH group and patients qualified for tonsillectomy or UPPP.

Patients Group	Mean Value VAS	Min Value VAS	Max Value VAS	SD
tonsillectomy	8.6	5.0	10.0	1.6
UPPP	8.2	5.0	10.0	1.8
PT	8.6	5.0	10.0	1.7
PTH	8.1	5.0	10.0	1.7

## Data Availability

The data analyzed in study are present in Department of Otolaryngology and Cranio-Maxillo-Facial Surgery and Department of Pathomorphology, Military Institute of Medicine—National Research Institute.

## Abbreviations

The following abbreviations are used in this manuscript:

QoL	quality of life
PT	palatine tonsillitis
PTH	palatine tonsillar hypertrophy
UPPP	uvulopalatopharyngoplasty
GBI	Glasgow Benefit Inventory
GBIgh	general health subscale of GBI
GBIph	physical health subscale of GBI
GBIss	social support subscale of GBI
VAS	Visual Analogue Scale

## Appendix A

Schwentner-modified GBI questionnaire:
